# How the Ectopic Expression of the Barley *F-Box* Gene *HvFBX158* Enhances Drought Resistance in *Arabidopsis thaliana*

**DOI:** 10.3390/ijms26010342

**Published:** 2025-01-02

**Authors:** Shuting Wen, Yicheng Chen, Xingzhe Yang, Guo Zhang, Lulu Jin, Xiaoqin Zhang, Yunxia Fang, Dawei Xue

**Affiliations:** 1 College of Life and Environmental Sciences, Hangzhou Normal University, Hangzhou 311121, China; stwen2468@163.com (S.W.); cyicheng2001@163.com (Y.C.); oliver00i@163.com (X.Y.); zhangguo@stu.hznu.edu.cn (G.Z.); 18057153961@163.com (L.J.); dwxue@hznu.edu.cn (D.X.); 2 Zhejiang Provincial Key Laboratory for Genetic Improvement and Quality Control of Medicinal Plants, Hangzhou Normal University, Hangzhou 311121, China

**Keywords:** *Hordeum vulgare* L., *Arabidopsis thaliana*, stress response, abiotic stress resistance

## Abstract

In this study, the drought-responsive gene *HvFBX158* from barley was transferred to *Arabidopsis thaliana*, and overexpression lines were obtained. The phenotypic characteristics of the transgenic plants, along with physiological indicators and transcription level changes of stress-related genes, were determined under drought treatment. Under drought stress, transgenic plants overexpressing *HvFBX158* exhibited enhanced drought tolerance and longer root lengths compared to wild-type plants. Additionally, malondialdehyde and hydrogen peroxide contents were significantly lower in transgenic lines, while superoxide dismutase activity was elevated. Quantitative RT-PCR showed that the expression levels of drought and stress response genes, including *AtP5CS, AtDREB2A, AtGSH1, AtHSP17.8*, and *AtSOD*, were significantly upregulated. Transcriptome analysis further confirmed that *HvFBX158* regulated multiple stress tolerance pathways. In summary, the overexpression of the *HvFBX158* gene enhanced drought tolerance in *Arabidopsis thaliana* by regulating multiple stress response pathways. This study provides a practical basis for improving drought-resistant barley varieties and lays a foundation for subsequent research on *F-box* family genes for stress resistance in barley.

## 1. Introduction

Due to global climate warming and frequent extreme weather events, the agricultural ecological environment has been severely damaged. Non-biological stress has emerged as a primary constraint on global crop production and poses a significant threat to food security [1,2]. According to statistics, in the past ten years, global crop production losses caused by drought have reached up to USD 30 billion [3]. Therefore, strengthening plant resistance to adverse conditions is crucial for sustainable agricultural development.

In response to non-biological stress, plants have evolved regulatory mechanisms to enhance their tolerance. Extensive researches have demonstrated the critical role of F-box proteins in plant growth and stress tolerance [4,5,6]. F-box proteins function as core components of the S-phase kinase-associated protein 1 (SKP1)-cullin 1 (CUL1)-F-box protein (SCF) complex and participate in the ubiquitin-mediated protein degradation pathways [7]. In this system, F-box proteins interact with SKP1 or its homologous region within the SCF complex through the F-box domain located at the N-terminus, thus mediating protein–protein interactions [8,9,10]. Additionally, certain conserved structural domains at the C-terminus facilitate the recruitment of F-box proteins to substrates under various stimuli [10,11], thereby regulating ubiquitination-mediated degradation pathways that positively or negatively modulate plant stress responses such as temperature, drought, and salinity stresses [5]. F-box proteins mediate plant tolerance to drought and salinity through multiple signaling pathways. For instance, the F-box protein FOF2 positively regulates plant drought tolerance via an abscisic acid (ABA)-dependent signaling pathway in *Arabidopsis thaliana* [6]. *MAX2* positively regulates drought and saline stress tolerance by modulating endogenous ABA signal transduction [12]. Furthermore, the *OsFBX257* gene responds to ABA signals, regulating drought tolerance, and the overexpression plants have significantly reduced leaf water loss [13]. The cuticle is an important structure in drought resistance. In *Arabidopsis thaliana*, *KUF1* negatively regulates plant drought tolerance by inhibiting cuticle formation, affecting the degree of stomatal closure and impacting root development [14]. *F-box* gene-mediated antioxidant effects can also affect a plant’s resistance to adverse conditions. For example, in wheat (*Triticum aestivum* L.), an overexpression of the *F-box* gene *TaFBA1* enhances resistance to drought by improving antioxidative capacity and water retention [15]. In *Arabidopsis thaliana*, the *F-box* gene *AtPP2-B11* enhances salt stress resistance by upregulating *AnnAt1* expression, inhibiting reactive oxygen species (ROS) accumulation, and maintaining Na^+^ homeostasis under salt stress conditions [16]. Moreover, *MAIF1* overexpression in rice (*Oryza sativa* L.) may reduce drought resistance, potentially by increasing cell division within roots [17].

Barley is the fourth most important cereal crop in the world, after wheat, maize (*Zea mays*), and rice. Unlike wheat, rice, and maize, barley predominantly grows in arid and semi-arid regions as well as marginal areas characterized by low and variable precipitation, and its yield is typically influenced by water scarcity and heat stress [18,19,20]. Therefore, improving the drought tolerance of barley has important practical significance for increasing its yield and quality, expanding its planting area, and enhancing its economic benefits [21]. However, studies related to *F-box* genes in plants primarily focus on rice [22], *Arabidopsis thaliana* [9], and wheat [23], with limited researches on the involvement of *F-box* genes under abiotic stress conditions, especially drought stress in barley.

In the early analyses of the barley *F-box* gene family, the expression of the *HvFBX158* gene was shown to be induced by drought stress [24]. To further explore its function, we transferred the *HvFBX158* (*HORVU. MoreX.r3.5HG0457960*) gene into *Arabidopsis thaliana*, and screened overexpression (OE) lines, so as to explore how the gene responds to drought through the determination of physiological indicators and regulatory pathways related to drought stress.

## 2. Results

### 2.1. Screening and Identification of Homozygous Transgenic Arabidopsis Lines

**Figure 1:**
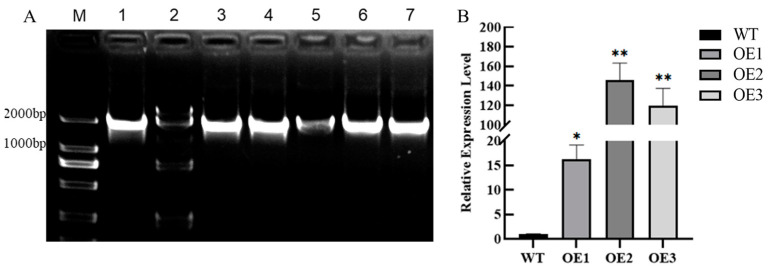
Identification of transgenic *Arabidopsis* lines. (**A**) PCR identification of T_3_ transgenic *Arabidopsis thaliana*, m: 2000 bp; 1: plasmid; 2: wild-type *Arabidopsis thaliana*; 3–7: transgenic *Arabidopsis*. (**B**) qRT-PCR detection of transgenic *Arabidopsis* was determined using the Student’s *t*-test, and the error bar represents the standard error (*n* ≥ 3, * *p* < 0.05; ** *p* < 0.01). WT and OE are the abbreviations of wild type and overexpression line, respectively.

We screened homozygous transgenic *Arabidopsis* strains using agar plates with Kanamycin, in which positive plants showed normal growth and had green leaves, while negative plants showed dysplasia and had pale yellow leaves (Supplementary Figure S1A–D). To further detect the presence of the *HvFBX158* gene in the T_3_ homozygous lines, we extracted DNA from both WT and transgenic strains and amplified the *HvFBX158* gene (1320 bp). Agarose gel electrophoresis showed that the other five transgenic lines had the same bright band as lane 1 of the positive control, and the sizes were consistent with the expectation except for lane 2 for WT (Figure 1A). Subsequently, three OE lines (OE1, OE2, and OE3) showing good growth and bright bands were selected for Quantitative RT-PCR (qRT-PCR) to detect the expression level of the *HvFBX158* gene. The detection results showed that the expression level of the *HvFBX158* gene in transgenic lines OE1, OE2, and OE3 was much higher than that in WT (Figure 1B). These three homozygous OE lines were used for subsequent drought stress experiments.

### 2.2. Changes in the Root Length of HvFBX158 Transgenic Arabidopsis at the Seedling Stage Under Drought Stress

**Figure 2:**
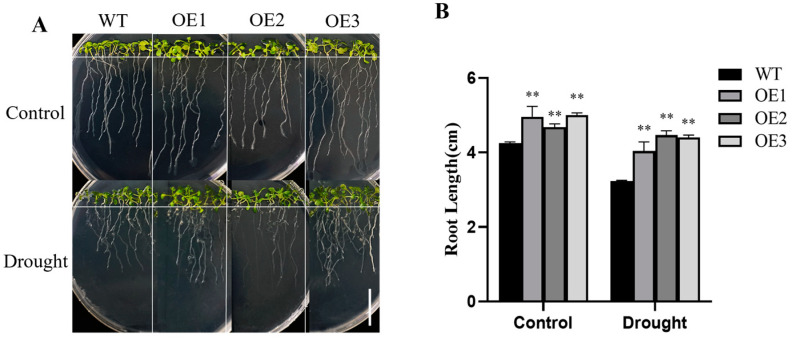
The drought resistance of *HvFBX158* transgenic *Arabidopsis* under drought stress. (**A**) Roots of the wild-type and transgenic *Arabidopsis* seedlings under normal (0% PEG 6000) and drought stress (6% PEG 6000) conditions (*n* ≥ 12); Scale bar = 3 cm. (**B**) Comparison of root length data of wild-type and transgenic *Arabidopsis* seedlings under drought stress. Data were analyzed by the Student’s *t*-test, and the error bar represents standard errors (*n* ≥ 3, ** *p* < 0.01). WT and OE are the abbreviations of wild type and overexpression line, respectively.

After observing the WT and *HvFBX158* transgenic lines, we found the root length of the OE lines was increased by 16.5%, 8.2%, and 17.6% compared with the WT, respectively. In the medium containing PEG 6000, the root growth of both WT and the OE lines was inhibited. However, after drought stress, the root length of WT was decreased by 23.75% compared with the control group, while the root length of OE1, OE2, and OE3 was decreased by 18.58%, 4.25%, and 12.05% compared with the control group, respectively (Figure 2). Based on these results, we preliminarily concluded that the *HvFBX158* gene enhanced the drought stress tolerance of transgenic *Arabidopsis*.

### 2.3. Phenotypic Characteristics at the Rosette Stage Under Drought Stress

**Figure 3:**
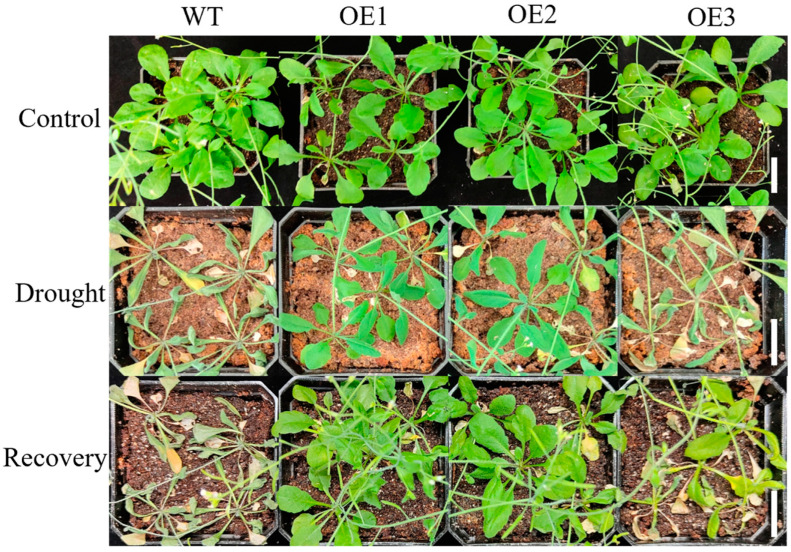
Phenotypes of wild-type and OE lines at the rosette stage under drought stress. WT and OE are the abbreviations of wild type and overexpression line, respectively. Control: wild-type and OE lines cultivated under normal conditions for 30 days; Drought: wild-type and OE lines after 21 days of drought treatment; Recovery: wild-type and OE lines after 3 days of rehydration after drought treatment. Scale bar = 2 cm. (Culture conditions of control group is 20–22 °C, the light cycle is 16/8 h, and the relative humidity is 60–70%; the drought treatment group was not watered.)

We observed *Arabidopsis* plants after their drought treatment and rehydration and found that under normal growth conditions, there were no significant differences in morphology or leaf number between WT and the OE lines. However, after drought treatment, both WT and the OE lines exhibited varying degrees of dehydration, leaf curling, wilting, and drying. Notably, these phenotypes appeared first and were more pronounced in WT compared to the OE lines. After 21 d of drought, the growth of the OE1 and OE2 lines was significantly better than WT. After 3 d of rehydration, the WT largely withered, whereas most seedlings of the OE1, OE2, and OE3 lines survived, and their leaves largely returned to their normal state (Figure 3). This suggested that transgenic plants exhibited a certain degree of drought stress tolerance.

### 2.4. Detection of Physiological and Biochemical Indicators

**Figure 4:**
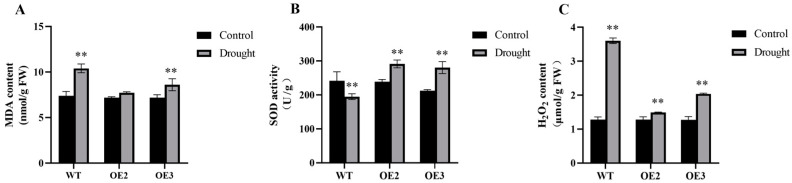
Physiological index data analysis of WT and OE lines under normal and drought treatment. (**A**) MDA content. (**B**) SOD enzyme activity. (**C**) H_2_O_2_ content. The data were analyzed by the Student’s *t*-test, and the error bar indicates the standard error (n ≥ 3, ** *p* < 0.01). WT and OE are the abbreviations of wild type and overexpression line, respectively. MDA, SOD, and H_2_O_2_ are abbreviations of malondialdehyde, superoxide dismutase, and hydrogen peroxide, respectively.

Leaves of the OE2, OE3, and WT were collected after 21 d of drought treatment, and the malondialdehyde (MDA) content, superoxide dismutase (SOD) enzyme activity, and hydrogen peroxide (H_2_O_2_) content of each group were measured separately. Under normal culture conditions, the MDA and H_2_O_2_ contents, as well as SOD activity, were essentially identical across WT, OE2, and OE3. Following drought treatment, the increase in MDA and H_2_O_2_ contents in WT was significantly greater than that in the transgenic lines. Specifically, the H_2_O_2_ content in WT plants had increased significantly, reaching 2.4 and 1.7 times higher than that observed in OE2 and OE3, respectively (Figure 4C). Additionally, SOD activity was significantly increased in both transgenic lines.

### 2.5. Expression of Stress-Related Genes in Arabidopsis thaliana Transformed with the HvFBX158 Under Drought Stress

**Figure 5:**
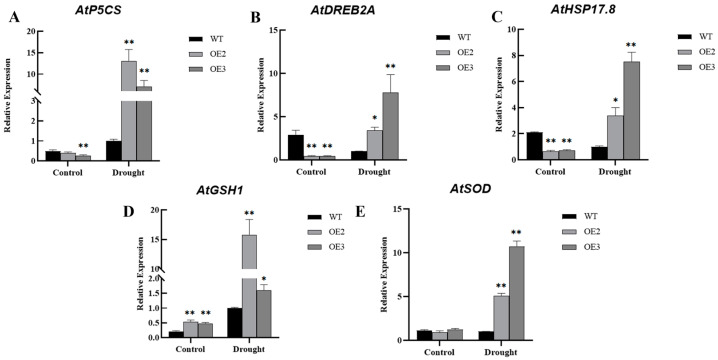
Expression analysis of stress-related genes under drought conditions. Relative expressions of *AtP5CS* (**A**), *AtDREB2A* (**B**), *AtHSP17.8* (**C**), *AtGSH1* (**D**), and *AtSOD* (**E**). The Student’s *t*-test was employed to ascertain significant differences, and the error bar indicated the standard error (*n* ≥ 3, * *p* < 0.05; ** *p* < 0.01). WT and OE are the abbreviations of wild type and overexpression line, respectively.

To investigate the regulatory pathways of *HvFBX158* in response to drought stress, we examined the expression levels of several stress-related genes, including osmosis-related gene *AtP5CS*, heat-tolerant transcription factor *AtDREB2A*, heat-shock protein *AtHSP17.8*, and oxidative damage-related genes *AtGSH1* and *AtSOD*. In the control group, the expression levels of *AtP5CS*, *AtDREB2A*, and *AtHSP17.8* genes in OE2 and OE3 showed a decreasing trend, while the expression level of *AtGSH1* was significantly elevated compared with WT. There was no significant change in *AtSOD* expression. After 21 d of drought treatment, the expression levels of *AtP5CS* and *AtGSH1* in WT, OE2, and OE3 were significantly upregulated, but the elevated expressions were more significant in the transgenic lines. For example, expressions of the *AtP5CS* and *AtGSH1* genes were 13 and 15.8 times higher, respectively, in OE2 than in WT after drought stress (Figure 5). Therefore, it can be preliminarily concluded that the *HvFBX158* gene increases the resistance of transgenic plants to drought stress by regulating the expression levels of stress-related genes, conferring stress tolerance to the plant.

### 2.6. Transcriptome Analysis of Differentially Expressed Genes Under Drought Stress

**Figure 6:**
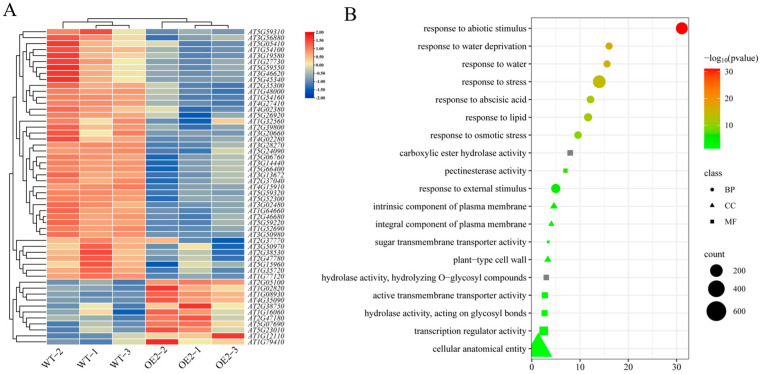
GO enrichment map of differentially expressed genes. (**A**) Cluster analysis of differentially expressed genes of WT and OE2 involved in GO entries in the dehydration response. (**B**) Bubble map of GO functional enrichment analysis of differentially expressed genes in WT vs. OE2. WT and OE are the abbreviations of wild type and overexpression line, respectively. BP, CC, and MF are abbreviations of biological processes, cellular components, and molecular functions, respectively. GO is the abbreviation of gene ontology.

Based on the expression levels of the relevant genes, we selected the OE2 strain for transcriptome sequencing to identify differentially expressed genes (DEGs) between the WT and OE line treated with drought stress. A total of 536 upregulated and 394 downregulated DEGs were detected (Supplementary Figures S2 and S3). According to the Gene Ontology (GO) enrichment analysis (Supplementary Figure S4), the biological processes (BP) categories were mainly enriched in non-biological stimuli (GO: 000928), water deprivation responses (GO: 0009414), and secondary metabolite biosynthetic processes. The DEGs in cellular components (CC) were mainly enriched in the intrinsic component of plasma membrane (GO: 0031226), integral component of plasma membrane (GO: 0005887), and cell wall (GO: 0005618). The molecular functions (MF) were mainly enriched in the following categories: oxidoreductase activity (GO: 0016491), secondary active transmembrane transporter activity (GO: 0015291), and hydrolase activity, hydrolyzing O-glycosyl compounds (GO: 0004553). Subsequently, we used the False Discovery Rate (FDR) value as the screening criterion to select the 20 most significantly enriched terms (Figure 6B), including signaling molecules related to plant stress responses, such as ABA [25], and the indole acetic acid (IAA)-related transferase signaling pathway [26]. Following a cluster analysis of the dehydration response items, 11 upregulated and 42 downregulated genes were significantly enriched in this category. The upregulated genes were associated with annexin pathways, sugar transport mechanisms, and transpiration pathways pertinent to multifocal osmosis regulation. Conversely, the downregulated genes were predominantly involved in pathways related to Ca^2+^ permeability, ABA signaling, glycoamino acid catabolism, lipid transfer processes, and the stabilization of intracellular proteins and enzymes, thereby mitigating protein aggregation [27] (Figure 6B). The expression levels of drought-tolerant genes increased in the OE lines. Drought-responsive genes, such as *AT1G08930* (*ERD6*), have been hypothesized to function as sugar transporters that actively respond to water scarcity [28]. Late embryogenesis abundant (LEA) proteins have been recognized for their positive role in abiotic stress responses and plant tolerance [29]. *LEA4*-*5* overexpression in *Arabidopsis thaliana* improves plant resilience against water deficiency. In contrast, knockout mutations affecting *LEA* subsets (*LEA4*-*2* and *LEA4*-*5*) resulted in a heightened susceptibility to dehydration—a phenomenon linked to the role of LEA proteins in maintaining the redox balance and cellular membrane stability under dehydrative conditions [30]. These findings indicate that most DEGs implicated in the water stress response were downregulated by OE lines during drought stress, further confirming that the *HvFBX158* gene plays a role in downregulating relevant genes under drought conditions.

## 3. Discussion

F-box proteins (FBXs) derive their name from a novel protein domain discovered in the cell cycle protein Cyclin F [31]. In plants, the *F-box* gene family boasts numerous members with significant variation [28,29]. Currently, multiple *F-box* genes have been cloned in plants, including *Arabidopsis thaliana*, rice, maize, and moss (*Hypnum plumaeforme*) [32,33,34,35]. The ubiquitin–proteasome system (UPS) is a crucial regulatory system by which F-box proteins degrade substrates in response to environmental stress. Genetic studies have demonstrated that a considerable number of cloned F-box proteins in plants form SCF complexes, participating in abiotic stress resistance through positive or negative regulation. Under normal conditions, F-box proteins recognize and ubiquitinate positive regulators of stress resistance, leading to their degradation, while active negative regulators shut down the stress response. Conversely, under adverse conditions, F-box proteins degrade positive regulators of the stress response, rendering them non-expressed, while non-degraded positive regulators trigger the stress response [36]. For instance, in *Arabidopsis thaliana*, F-box proteins EBF1 and EBF2 directly interact with phytochrome interacting factor 3 (PIF3), which negatively regulates chilling stress, mediating the hydrolysis of PIF3 by the 26S proteasome and enhancing chilling resistance [37]. *RIFP1* negatively regulates the expression of ABA response genes by degrading the ABA receptor RCAR3, negatively regulating ABA signaling transduction, and enhancing the drought resistance of *rifp1* [38]. Acting as a negative regulator, the At1g08710 protein interacts with the ADA2b protein to regulate plant growth and resist drought stress [39]. Based on our preliminary analysis of the barley *F-box* gene family, the expression of the *HvFBX158* gene was shown to be induced by drought stress. To further investigate the mechanism by which this gene responds to drought stress, we transferred the *HvFBX158* gene into *Arabidopsis thaliana* and analyzed its function.

To determine the expression of the *HvFBX158* gene in various tissues, we utilized qRT-PCR to detect the expression of the barley *HvFBX158* gene. The *HvFBX158* gene was expressed in all tested tissues, but the expression level was higher in young leaves and lower in young roots, spikes, and leaves (Supplementary Figure S5). Due to the long cycle and low success rate of barley transgenic crops, we transferred the *HvFBX158* gene into the model organism *Arabidopsis thaliana* for phenotypic observation. After 21 d of drought and 3 d of rehydration, the degree of leaf wilting and the survival rate of the transgenic plants were significantly superior to those of wild-type plants (Figure 3), and the *HvFBX158* expression after the drought treatment increased compared to that before treatment (Supplementary Figure S6). Based on these results, we speculated that the *HvFBX158* gene enhances the drought resistance of transgenic *Arabidopsis thaliana* plants.

Drought-resistant plants often exhibit significant differences in physiological indicators compared to WT plants. MDA is an important indicator for measuring membrane lipid peroxidation, indirectly reflecting the degree of membrane system damage and the plant’s stress resistance [40]. Plants produce ROS under environmental stress and other factors. The level of ROS accumulation is often characterized by the relatively stable H_2_O_2_, which is therefore considered the main form of ROS involved in cell signaling [41,42]. SOD plays a role in plant cells under ROS damage [41,43]. To further investigate the function of the *HvFBX158* gene, we evaluated relevant physiological and biochemical indicators and found that transgenic *Arabidopsis thaliana* showed a decrease in MDA content, which measures oxidative damage, an increase in antioxidant enzyme SOD activity, and a significant decrease in H_2_O_2_ content, which represents ROS accumulation, under drought stress compared to WT (Figure 4). These results indicated that transgenic *Arabidopsis thaliana* with the *HvFBX158* gene has an enhanced antioxidant defense ability, clears excessive H_2_O_2_, reduces MDA accumulation, and thereby resists drought stress. Based on the differences in various drought stress phenotypes and their corresponding physiological indicators, we concluded that the *HvFBX158* gene improves the drought tolerance of *Arabidopsis thaliana*.

The drought resistance response mechanism in plants is complex and regulated by multiple genes and metabolic pathways. In recent years, research on plant drought resistance mechanisms has gradually increased, and drought resistance genes have been identified [44,45,46]. Drought initially causes osmotic stress, which triggers secondary damage, such as oxygen metabolism disorders [47,48]. Under osmotic stress, plants accumulate proline (Pro), sugars, and various organic compounds to reduce osmotic potential and improve cell water retention [49]. Among these, Pro can scavenge ROS under osmotic stress conditions and stabilize proteins, membranes, and subcellular structures by maintaining intracellular and extracellular osmotic pressure homeostasis [50]. P5CS is a key enzyme in the Pro synthesis pathway in plants. Plants tolerate and resist drought stress by regulating Pro synthesis. Quantitative analysis showed that, under drought stress, the expression levels of *AtP5CS* in both WT and the OE line significantly increased, but the expression level of *AtP5CS* in the OE line was significantly higher than WT, indicating that the osmotic adjustment system plays an important role in resisting drought stress. The antioxidant system often accompanies the drought response mechanism, both protecting cells from the damage caused by free radicals using small molecules and protective enzymes, such as glutathione (GSH), SOD, and catalase (CAT) [51]. In *Arabidopsis thaliana,* AtGSH1 and AtSOD are key enzyme genes in the GSH and SOD synthesis pathways, respectively, playing crucial roles in protecting cells from oxidative stress, the redox regulation of enzymes, and defense against ROS oxidative damage [52,53]. The analysis revealed that the expression levels of both *AtGSH1* and *AtSOD* were significantly increased after drought treatment, indicating that both enzymatic and non-enzymatic systems within the plant function to scavenge ROS, consistent with the results of the SOD enzyme activity assay. Small heat-shock proteins (sHSPs) are ubiquitous in plants and have been demonstrated to exhibit chaperone activity, preventing the formation of non-specific protein aggregates in response to adverse environmental conditions, such as dehydration and drought stress [54,55]. AtHSP17.8, a member of the CI-type cytoplasmic sHSPs, positively regulates plant drought resistance [56]. Dehydration-responsive element-binding protein 2A (DREB2A) functions as a key transcription factor for heat and drought tolerance [57,58], and its overexpression enhances plant drought tolerance. Based on researches related to drought-responsive genes, we selected five stress-related genes, namely *AtP5CS*, *AtDREB2A*, *AtGSH1*, *AtHSP17.8*, and *AtSOD*, for experimental verification after drought treatment in transgenic lines (Figure 5). The expression levels of these drought-responsive genes were also significantly upregulated in the OE lines of *Arabidopsis thaliana* transformed with the *HvFBX158* gene. Based on these experimental results, it is speculated that the *HvFBX158* gene responds to drought stress by regulating physiological and metabolic pathways.

In the early stage of the analysis of the barley *F-box* gene family, the expression of the *HvFBX158* gene was shown to be induced by drought stress [24]. To further explore the molecular mechanism of *HvFBX158*, this study used transcriptome sequencing technology to analyze WT and *HvFBX158 Arabidopsis thaliana* lines after drought treatment. An analysis of the DEGs revealed that after drought treatment, the *Arabidopsis thaliana* OE line had 536 upregulated and 394 downregulated DEGs compared to WT (Supplementary Figure S2). GO analysis showed that DEGs were mainly enriched in ABA, amino acid metabolism, plasma membrane, and hydrolase activity (Figure 6). After drought treatment, relevant genes (Figure 6A), such as *KCS3*, which functions as a negative regulator of wax metabolism by reducing the enzyme activity of KCS6, were downregulated, conferring drought tolerance [59]. *AtPUB18* functions as a negative regulator to regulate ABA-mediated stomatal closure and the water stress response [60]. Based on the enrichment of GO terms, we speculated that the *HvFBX158* gene confers drought tolerance to plants by downregulating genes related to sugar metabolism and hormone signaling.

In this study, we heterologously expressed the barley gene *HvFBX158* in *Arabidopsis* and obtained overexpression lines. By observing changes in the phenotype, physiological indicators, and related drought-responsive genes under drought stress and comparing transcriptome data, overexpression of the *HvFBX158* gene was shown to improve drought resistance, thus providing a basis for improving drought tolerance in barley varieties. However, the specific regulatory mechanism and action pathway through which this gene operates have not been studied under drought stress, and due to the time-consuming and inefficient process of creating transgenic barley plants, there is no conclusive evidence of its effects in barley. In the future, we aim to further explore the available regulatory pathways identified from the transcriptome to promote innovation in barley and other crops.

## 4. Materials and Methods

### 4.1. Plant Materials

The barley variety ‘Morex’ was used as the experimental material. The seeds were germinated in the dark at 24 °C for 48 h and then transferred to 96-well plates for hydroponic culture in Hoagland’s solution at 24 °C, with a light/dark cycle of 14/10 h.

The ‘Columbia’ (Col-0) ecotype of *Arabidopsis thaliana* was used as the WT. All plants were grown at 20–22 °C with a 16/8 h light cycle and a 60–70% relative humidity.

### 4.2. Overexpression Vector Construction and Transgenic Plant Identification

We designed primers by Primer Premier v5.0 (PREMIER Biosoft, San Francisco, CA, USA) to amplify the full-length coding sequence of the *HvFBX158* gene, and the target gene was ligated into the *pCAMBIA2300-35s-egfp* vector using the T4 DNA ligase. PCR identification and sequencing were performed. *Agrobacterium tumefaciens* was transformed by electroporation. The recombinant expression vector was transformed into seeds of *Arabidopsis thaliana*, namely wild-type ecotypes Col-0 by the inflorescence impregnation method, and T_1_ seeds were harvested. T_1_ seedlings were screened on a half Murashige and Skoog (1/2 MS) medium (1.5% sucrose and 0.8% agar) containing 50 ng/µL kanamycin [61]. Normally growing plants were selected for individual seed collection, and T_2_ seedlings meeting the separation ratio of 3:1 were screened again with antibiotics. A homozygous T_3_ line containing the inserted gene was subsequently identified by kanamycin. Finally, we detected the expression of the *HvFBX158* gene by qRT-PCR [62,63]. All primers are listed in Supplementary Table S1.

### 4.3. Phenotype Observation After Drought Stress Treatment

#### 4.3.1. *Arabidopsis* Seed Germination and Drought Stress Treatment

The WT and transgenic *Arabidopsis* seeds were surface disinfected (10% sodium hypochlorite for 3 min, followed by rinsing with absolute ethanol, cleaning 3-5 times for 2 min) and sown on a 1/2 MS medium, and a 1/2 MS medium containing 6% PEG 6000 [61], and cultured vertically for 10 d. *Arabidopsis* seedlings were photographed after 10 d, and their root length was determined.

#### 4.3.2. Stress Treatment of *Arabidopsis* Plants at the Rosette Stage

WT and transgenic *Arabidopsis* seeds were sown and watered once every 3 d during the growth period. Watering was ceased after 4 weeks, while all other growth conditions remained unchanged [30]. After 21 d, the growth was photographed and recorded, and the plants were rehydrated. After 3 d of rehydration, their growth was again observed and photographed.

### 4.4. Extraction of Total RNA and qRT-PCR Analysis

The total RNA was extracted from the aboveground parts of the *Arabidopsis thaliana* seedlings subjected to drought stress for 18 d using the Eastep^®^ Super Total RNA Extraction Kit (Promega, Shanghai, China), and the resulting product was stored in an ultra-low temperature freezer for subsequent experiments. The qRT-PCR results were calculated using the 2^−ΔΔCt^ (Livak method), and three biological replicates were performed for each sample. The primers used in the experiment are listed in Supplementary Table S1.

### 4.5. Determination of MDA, H_2_O_2_, and SOD Enzyme Activities

The content of the MDA, H_2_O_2_, and SOD enzyme activity were determined in the leaves of WT and transgenic plants subjected to drought stress using reagent kits provided by Suzhou Grace Biotechnolgy Co., Ltd. (Suzhou, China), following the manufacturer’s instructions.

### 4.6. Transcriptome Sequencing Analysis

After 4 weeks of standard culture, *Arabidopsis thaliana* was subjected to drought treatment for 18 days, and then samples of WT and OE2 lines were collected. For each line, six leaves of relatively uniform size were sampled for each biological replicate, resulting in a total of three replicates. Transcriptome sequencing was conducted by Shanghai Personal Biotechnology Co., Ltd. (Shanghai, China). The data analysis charts of transcriptome in the article (Figure 6, Supplementary Figure S4) were plotted by https://www.bioinformatics.com.cn (last accessed on 24 September 2024), an online platform for data analysis and visualization [64].

### 4.7. Statistical Analysis of Experimental Data

This study used GraphPad Prism 9.5.0 for Windows (GraphPad Software, San Diego, California USA, www.graphpad.com) software and SPSS 26.0 (IBM, Armonk, NY, USA) software to create bar charts and conducted significant difference tests by Student’s *t*-test.

## 5. Conclusions

In this comprehensive study, we heterologously expressed the barley gene *HvFBX158* into *Arabidopsis thaliana* and successfully generated OE lines. Through phenotypic observation, physiological indicators measurement, transcriptome analysis, and quantitative verification of the drought-response genes under the drought condition, we believe that the *HvFBX158* gene is likely to be induced by drought stress. Moreover, the overexpression of this particular gene has the potential to significantly improve the drought resistance of transgenic *Arabidopsis* lines. Consequently, this finding provides a solid and reliable basis for enhancing the drought tolerance of barley varieties, opening up new possibilities for future research and practical applications in the field of agricultural genetics.

## Data Availability

Data is contained within the article and Supplementary Materials.

## Supplementary Materials

The following supporting information can be downloaded at https://www.mdpi.com/article/10.3390/ijms26010342/s1.
