# Supplementary material for: How the Ectopic Expression of the Barley F-Box Gene HvFBX158 Enhances Drought Resistance in Arabidopsis thaliana
Source: Int J Mol Sci. 2025 Jan 2;26(1):342. doi: 10.3390/ijms26010342 (PMC11719962; doi:10.3390/ijms26010342)
Supplement: Supplementary file 1 [file ijms-26-00342-s001.zip › ijms-3384861-supplementary.pdf]

Table S1. Primers designed for vector construction and qRT-PCR analysis

Primer Name	Forward primer sequence	Reverse primer sequence
HvFBX158	5′ATGTCGTTTCGTAGCATTGTGCGTGATGT 3′	5′TCATTCGCAAGCCAGCTTTGTGTCGAA3′
HvFBX158-test	5′CGTCGCCGTCCAGCTCGACCAGG3′	5′CATGGTCCTGCTGGAGTTCGTGA3′
HvFBX158-eGFP	5′TCTAGAGGATCCCCGGGATGTCGTTTCGTAG CATTGTGCGTGATGT3′	5′GAGCTCGGTACCCGGGTCATTCGCAAGCC AGCTTTGTGTCGAA3′
AtDREB2A	5′CTACAAAGCCTCAACTACGGAATAC3′	5′AAACTCGGATAGAGAATCAACAGTC3′
AtGSH1	5′ATCTACGCTTTGTCCCCATTC3′	5′AAACTCGGATAGAGAATCAACAGTC3′
AtHSP17.8	5′GTGGAGATAGAGGACGACGACAGTG3′	5′CTTAACTTGAGCCTTCTTCTTAGC3′
AtSOD	5′CGCATGATCCTTTGGCTTCG3′	5′ATGGATCCTGGTTGGCTGTG3′
qrt-FBX158	5′GCCGTTGCTCATTCCCTT 3′	5′TGCCTGGCCTCCATTCCT 3′
Actin	5′TGGATCGGAGGGTCCATCCT3′	5′GCACTTCCTGTGGACGATCGCTG3′

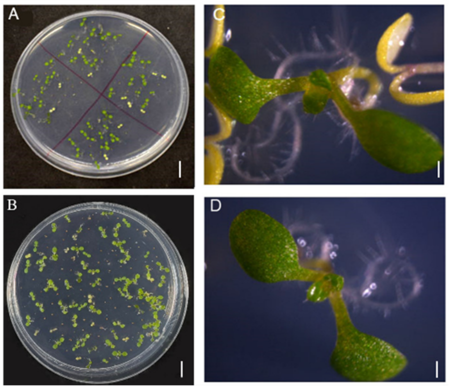

Figure S1. Screening and identification of transgenic Arabidopsis homozygous lines. (**A, B**) Media screening of T_2 generation transgenic Arabidopsis; Scale bar = 1 cm. (**C, D**) Morphology of T_2 and T_3 generation transgenic Arabidopsis under a microscope; Scale bar = 0.1 cm.

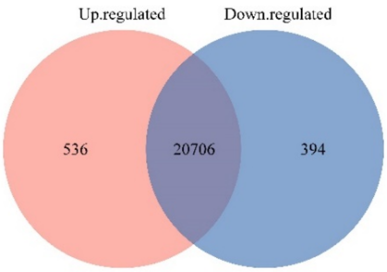

Figure S2. Number of differential genes between WT and OE after drought stress. WT and OE are the abbreviations of wild type and overexpression line, respectively.

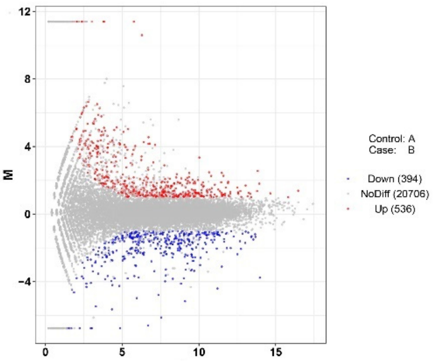

Figure S3. Volcano plot of differential genes between WT and OE after drought stress. WT and OE are the abbreviations of wild type and overexpression line, respectively.

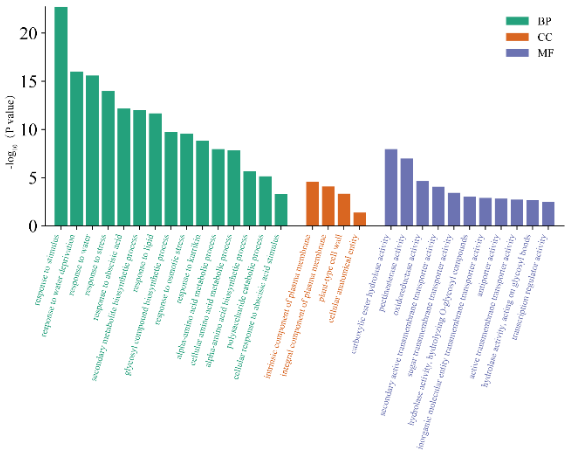

Figure S4. Histogram of GO functional enrichment analysis of differentially expressed genes after comparison between WT and OE2. WT and OE are the abbreviations of wild type and overexpression line, respectively. BP, CC and MF are abbreviations of biological processes, cellular components and molecular functions, respectively. GO is the abbreviation of gene ontology.

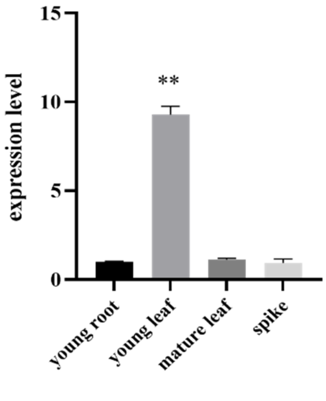

Figure S5. Tissue specific expression analysis of *HvFBX158* gene. Significant differences were etermined using a t-test (** $P < 0.01$). The error bar represents the standard error.

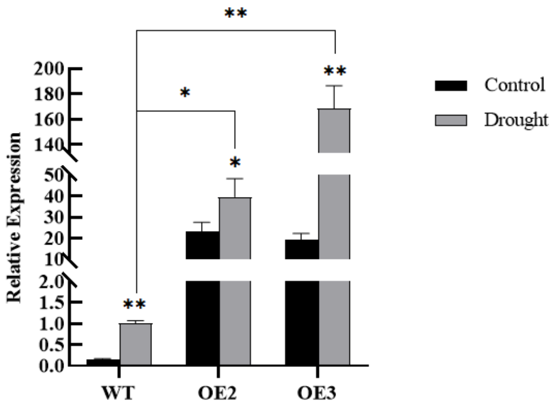

Figure S6. Comparison of HvFBX158 gene expression in WT, OE2 and OE3 before and after drought stress. Significant differences were etermined using a t-test (** $P < 0.01$). The error bar represents the standard error. WT and OE are the abbreviations of wild type and overexpression line, respectively.
